# Construction of a metastasis-associated ceRNA network reveals a prognostic signature in lung cancer

**DOI:** 10.1186/s12935-020-01295-8

**Published:** 2020-06-03

**Authors:** Qing Cao, Zewen Dong, Shuzhen Liu, Guoyan An, Bianbian Yan, Lei Lei

**Affiliations:** Key Laboratory of Resource Biology and Biotechnology in Western China, Ministry of Education, School of Medicine, Northwest University, Taibai North Road 229, Xi’an, 710069 Shaanxi China

**Keywords:** Lung cancer, Metastasis, ceRNA, lncRNA, LINC01010

## Abstract

**Background:**

Lung cancer is the most common cancer worldwide, and metastasis is the leading cause of lung cancer related death. However, the molecular network involved in lung cancer metastasis remains incompletely described. Here, we aimed to construct a metastasis-associated ceRNA network and identify a lncRNA prognostic signature in lung cancer.

**Methods:**

RNA expression profiles were downloaded from The Cancer Genome Atlas (TCGA) database. Gene Ontology (GO), Kyoto Encyclopedia of Genes and Genomes (KEGG) pathway analyses and gene set enrichment analysis (GSEA) were performed to investigate the function of these genes. Using Cox regression analysis, we found that a 6 lncRNA signature may serve as a candidate prognostic factor in lung cancer. Finally, we used Transwell assays with lung cancer cell lines to verify that LINC01010 acts as a tumor suppressor.

**Results:**

We identified 1249 differentially expressed (DE) mRNAs, 440 DE lncRNAs and 26 DE miRNAs between nonmetastatic and metastatic lung cancer tissues. GO and KEGG analyses confirmed that the identified DE mRNAs are involved in lung cancer metastasis. Using bioinformatics tools, we constructed a metastasis-associated ceRNA network for lung cancer that includes 117 mRNAs, 23 lncRNAs and 22 miRNAs. We then identified a 6 lncRNA signature (LINC01287, SNAP25-AS1, LINC00470, AC104809.2, LINC00645 and LINC01010) that had the greatest prognostic value for lung cancer. Furthermore, we found that suppression of LINC01010 promoted lung cancer cell migration and invasion.

**Conclusions:**

This study might provide insight into the identification of potential lncRNA biomarkers for diagnosis and prognosis in lung cancer.

## Background

Lung cancer is the most common cancer worldwide and the leading cause of cancer-related death in men and the second in women [1, 2]. In recent years, several studies have shown that abnormalities in noncoding genes are associated with lung cancer pathogenesis [3–6], but the mechanism whereby noncoding genes affect lung cancer metastasis remains incompletely understood.

The ENCODE (Encyclopedia of DNA Elements) Consortium revealed that less than 2% of the human genome is comprised of protein coding genes, while a dominant portion of transcripts are noncoding genes, which includes long noncoding RNAs (lncRNAs), pseudogenes and microRNAs (miRNAs) [7–9]. LncRNAs used to be considered transcriptional noise that have no biological function. Recently, increasing studies have revealed that lncRNAs are involved in many cellular processes, such as myocyte differentiation, immune response, cancer cell metastasis, proliferation, and drug resistance [10–12]. For instance, overexpression of lncRNA HAND2-AS1 inhibited migration of non-small cell lung cancer cells by downregulating TGF-β1 [13]. Furthermore, Fang et al. reported that lncRNA HOTAIR affects chemoresistance by regulating HOXA1 methylation in small cell lung cancer [14].

MiRNA is an endogenous small non‐coding RNA that also plays an important biological role in the development and metastasis of lung cancer [15]. Recently, Salmena et al. proposed the competitive endogenous RNA (ceRNA) hypothesis in which lncRNAs are able to regulate mRNAs expression as “miRNA sponges” by preferentially occupying the miRNAs response elements [16]. Kumar et al. demonstrated that Hmga2 promotes lung cancer progression by operating as a ceRNA for the let-7 miRNA family [17]. Moreover, PVT1 promotes expression of HIF-1α by functioning as a ceRNA for miR-199a-5p in Non-small cell lung cancer [18]. Therefore, construction of a ceRNA network could provide new perspectives for evaluating cancer regulatory networks.

In this study, we analyzed genomic data along with clinical information from The Cancer Genome Atlas (TCGA). Next, we used bioinformatics tools to construct a metastasis-associated lncRNAs-miRNAs‐mRNAs ceRNA network in lung cancer. Using multivariate Cox regression analysis, we discovered that a signature based on 6 lncRNAs may serve as an independent prognostic factor in lung cancer. Furthermore, we validated LINC01010 as a tumor suppressor lncRNA and might be involved in lung cancer ceRNA by competing with miR-372. This study reveals a ceRNA network in metastatic lung cancer, which may provide a useful basis for formulating early diagnosis and individualized treatments.

## Materials

### Data collection and differential expression analysis

Patient sample data sets and clinical information were extracted from TCGA database (https://portal.gdc.cancer.gov/) using the Genomic Data Commons (GDC) data transfer tool, including 32 lung cancer metastasis (M1) cases and 741 lung cancer nonmetastatic (M0) cases. We then used the “edgeR” package in R software to identify differentially expressed (DE) mRNAs, lncRNAs and miRNAs between M1 and M0 groups with thresholds of |logFC| > 1 and FDR < 0.05 [19]. All analyses were performed using R version 3.3.2.

### Functional enrichment analysis

To better understand the function of DE genes, we divided 1249 DE mRNAs into upregulated and downregulated groups for GO (Gene Ontology) analysis and KEGG (Kyoto Encyclopedia of Genes and Genomes) pathway analysis. We analyzed GO biological processes using DAVID (https://david.ncifcrf.gov/) and KEGG pathways using KOBAS (http://kobas.cbi.pku.edu.cn/). The top 15 pathways of GO and KEGG analysis were visualized using the R package ggplot2.

Considering that the correlation between LINC01010 and overall survival is the most significant among the 6 lncRNA signal, we further investigated the function of LINC01010. We searched for mRNAs co-expressed with LINC01010 using the Multi-Experiment Matrix (MEM) resource (https://biit.cs.ut.ee/mem/). Then, the top 200 mRNAs co-expressed with LINC01010 were selected for GO and KEGG pathway enrichment analysis.

Since LINC01010 was negatively correlated with hsa-mir-372, we assumed that LINC01010 may act as a ceRNA for hsa-mir-372. To confirm this hypothesis, we further studied the function of has-miR-372 and LINC01010. Lung cancer gene sets were downloaded from TCGA database and used to perform gene set enrichment analysis (GSEA). The phenotype label comprised groups with high-levels of has-miR-372 or LINC01010 versus those with low levels of has-miR-372 or LINC01010. *P*-values were used to estimate the statistical significance of enrichment scores.

### Constructing the competitive endogenous RNA network

**Fig. 1:**
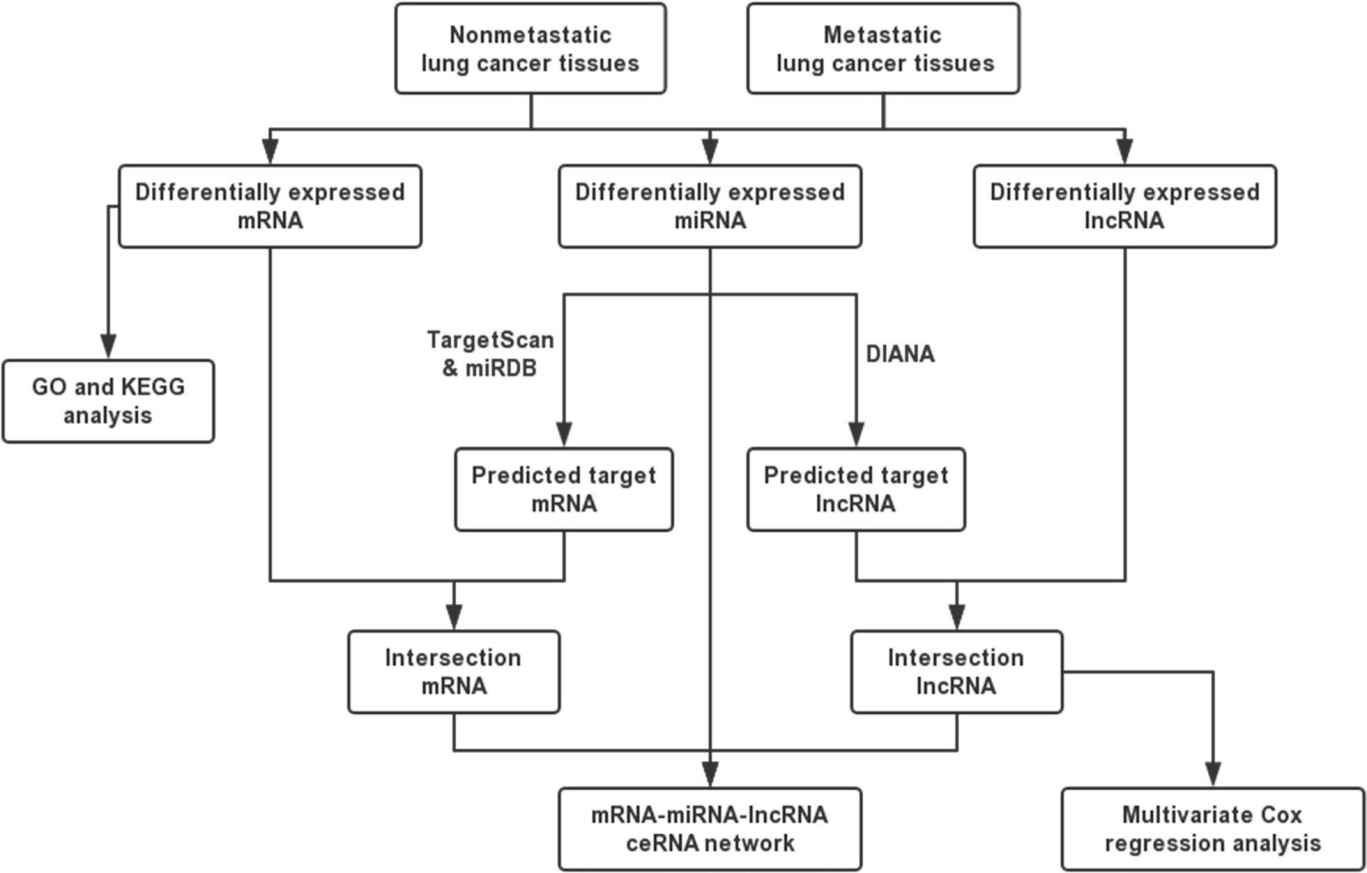
Flow chart of construction of the metastasis-associated ceRNA network in lung cancer

We constructed a ceRNA network containing DE mRNAs, miRNAs, and lncRNAs using bioinformatics tools. TargetScan (http://www.targetscan.org/) and miRDB (http://www.mirdb.org/) were used to predict mRNA-miRNA interactions. Then, DIANA was used to predict lncRNA-miRNA interactions. The predicted intersection results were retained to construct the ceRNA network (Fig. 1), which was visualized using Cytoscape v3.6.1. The network topologies were analyzed using the plug‐in NetworkAnalyzer tool in Cytoscape [20].

### Prognosis and survival curve analysis

Multivariate COX proportional hazards regression analysis was performed to identify a prognostic model of the lncRNAs in the ceRNA network. Mathematical models were established based on the Akaike Information Criterion (AIC) using the “Survival” R package [21]. We calculated the prognostic risk score as follows:

$$ \begin{aligned} {\text{Risk Score}} & =  \left( {0.0 6 6 2} \right) \, *{\text{LINC}}0 1 2 8 7+ \, \left( {0.0 9 7 8} \right) \, *{\text{ SNAP25}} - {\text{AS1}} \hfill \\ & \quad + \, \left( {0.0 5 6 7} \right) \, *{\text{LINC}}00 4 70 + \, \left( { - 0.0 7 4 5} \right) \, *{\text{AC1}}0 4 80 9. 2\hfill \\ & \quad + \, \left( { - 0. 20 1 8} \right) \, *{\text{LINC}}00 6 4 5+ \, \left( { - 0.0 7 2 6} \right) \, *{\text{LINC}}0 10 10 \hfill \\ \end{aligned} $$

All lung cancer patients were divided into low‐risk and high‐risk groups based on the median risk score. Overall survival (OS) curves of the two groups were generated using Kaplan–Meier analysis. The predictive value of the prognostic model over 5 years was evaluated by time-dependent receiver operating characteristic (ROC) curve analysis using the “survival ROC” R package [22].

### Cell culture and siRNA transfection

Human lung cancer cell lines (SPC-A-1and A549) were purchased from American Type Culture Collection (ATCC). SPC-A-1 and A549 cells were cultured in Roswell Park Memorial Institute 1640 medium and Dulbecco’s Modified Eagleʼs Medium (HyClone), respectively, supplemented with 10% fetal bovine serum (Gibco, Rockville, MD) at 37 ℃ and 5% CO_2_. A small interfering RNA for LNC01010 (siLNC01010) and a negative control (siRNA‐NC) were used in knockdown function experiments. 50 nM siRNA‐NC and 50 nM siLNC01010 (GenePharma, China) were transfected into A549 and SPC-A-1 cells using X-treme GENE siRNA Transfection Reagent (Roche, USA). Forty-eight hours after transfection, cells were harvested for RNA extraction or other functional experiments. SiRNA sequences are as follows: siLINC01010-1, 5′‐GCUGUUUGCUGGCAACAAATT‐3′; siLINC01010-2, 5′‐GCUGUUUGCUGGCAACAAATT‐3′; siLINC01010-3, 5′‐GCAGCAAAUGUAGAAACAUTT‐3′.

### RNA isolation and quantitative real time-PCR

Total RNA from cell samples was extracted using the TRIzol (Invitrogen, Carlsbad, CA) Pyrolysis method. Total RNA was reverse transcribed into complementary DNA using a Prime Script RT reagent kit (Takara, Tokyo, Japan). BestarTM qPCR MasterMix (DBI Bioscience) was used to perform qRT‐PCR on a CFX96 Touch Real‐Time PCR Detection System (Bio‐Rad, Hercules, CA). The following primers were used in qRT‐PCR: LNC01010 forward, 5ʹ-AATGATGCGGCTGAACAA-3ʹ, and reverse, 5ʹ-CCTTGGCTTGCCTATTACC-3ʹ; GAPDH forward, 5ʹ-TGCAAATCCCATCACCATCT-3ʹ, and reverse 5ʹ-TGGACTCCACGACGTACTCA-3ʹ. GAPDH was used as an endogenous control. qRT‐PCR conditions were as follows: 95 °C for 3 min, followed by 40 cycles of 95 °C for 10 s and 60 °C for 34 s with a melt curve from 60 to 95 °C.

### Cell migration and invasion assays

Cell migration and invasion abilities were evaluated using Transwell assays (Corning, MA, USA). 5 × 10^4^ cells were suspended in the upper chamber, and 700 μl medium containing 10% FBS was placed into the lower chamber. After 24 h incubation, A549 and SPC-A-1 cells that had migrated through the membrane were fixed with methanol for 15 min and stained with 10% crystal violet. For invasion assays, Matrigel (BD Biosciences, Bedford, MA, USA) was added into the upper chambers 12 h before the experiments. The number of cells were quantified in six random fields, and three independent experiments were performed.

### Cell proliferation assay

Cell proliferation ability was evaluated using a Cell Counting Kit-8 (CCK8) assay. CCK8 assay was conducted as previously described [23].

### Statistical analysis

Unpaired t-test (two-tailed) was used to analyze different groups of cellular experiments in GraphPad Prism 6 (GraphPad Software, Inc., San Diego, CA). Pearson’s test was used to measure the correlation between expression of LINC01010 and miR-372. *P* < 0.05 was considered statistically significant.

## Results

### Differentially expressed lncRNAs, miRNAs and mRNAs

To construct the metastasis-associated ceRNA network in lung cancer, we downloaded RNA expression profiles from TCGA database, which included 773 lung cancer samples. The differentially expressed (DE) mRNAs, lncRNAs and miRNAs between 32 metastatic tumor tissues and 741 nonmetastatic tumor tissues were explored using the “edgeR” package in R software. With the criteria of |logFC| > 1 and FDR < 0.05, we identified 1249 DE mRNAs (569 upregulated and 680 downregulated), 440 DE lncRNAs (221 upregulated and 219 downregulated) and 26 miRNAs (21 up-regulated and 5 downregulated) between nonmetastatic and metastatic tumor tissues. The results indicate that aberrant expression of these genes might be involved in lung cancer migration.

### GO and KEGG pathway analysis of DE genes

**Fig. 2:**
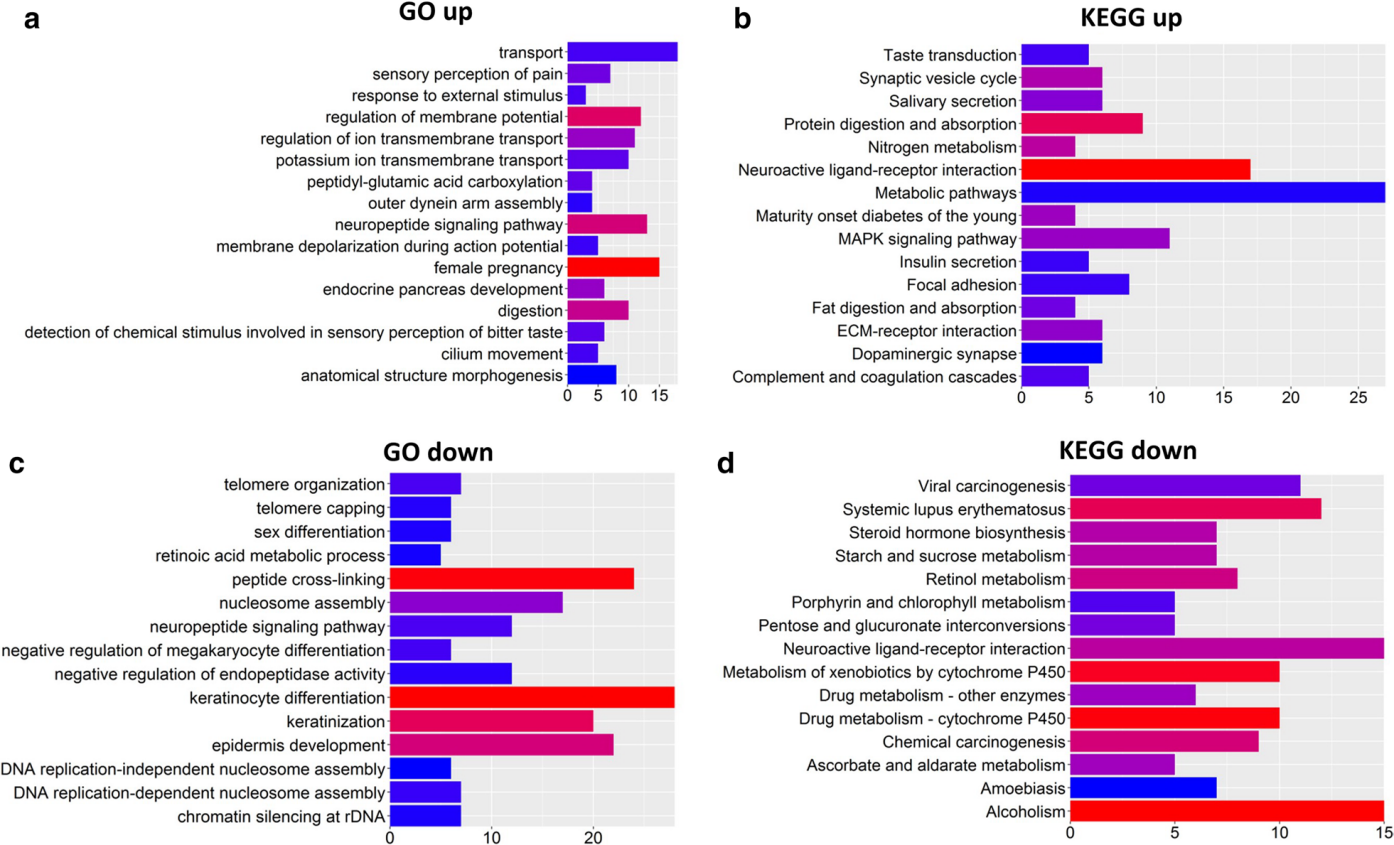
The functions of DE mRNAs between nonmetastatic and metastatic lung cancer tissues. **a**, **b** Top 15 GO and KEGG pathways of upregulated mRNA, respectively. **c**, **d** Top 15 GO and KEGG pathways of downregulated mRNA, respectively. Color from blue to red indicates − log_10_(*P*-value) from low to high. X-axes represent the number of genes involved in each pathway

To better understand the function of the identified DE genes, DE mRNAs were divided into upregulated and downregulated groups for analysis by DAVID and KOBAS bioinformatics resources. The top 15 GO biological processes and KEGG pathways of dysregulated genes are shown in Fig. 2 Among the top 15 GO and KEGG pathways of upregulated mRNAs (Fig. 2a, b), “GO:0006810 ~ transport”, “GO:0003341 ~ cilium movement”, “MAPK signaling pathway”, “ECM-receptor interaction” and “focal adhesion” are reported to promote invasion and metastasis in cancer [24, 25]. Downregulated genes participated in “GO:0031424 ~ keratinization”, “GO:0008544 ~ epidermis development” and “drug metabolism-cytochrome P450” (Fig. 2c, d), which reportedly inhibit invasion and metastasis of cancer [26, 27]. These results suggest that these above pathways may play important roles in lung cancer metastasis.

### Construction of the ceRNA network

To construct the metastasis-associated ceRNA network, we predicted interactions among DE mRNAs, miRNAs and lncRNAs using bioinformatics tools. Target mRNAs of DE miRNAs were predicted using TargetScan and miRDB, and the intersection of these two databases was selected. After we discarded target mRNAs that were not among DE mRNAs, 117 mRNAs (22 upregulated and 95 downregulated) were used to construct the ceRNA network (Additional file 1: Table S1). Next, we utilized DIANA to predict interactions between DE lncRNAs and DE miRNAs. We found the 22 miRNAs that are predicted to interact with 23 lncRNAs (Additional file 1: Table S2). Ultimately, 23 lncRNAs (20 downregulated and 3 upregulated) (Additional file 1: Table S3) and 22 miRNAs (4 downregulated and 18 upregulated) (Additional file 1: Table S4) were included in the ceRNA network.

**Fig. 3:**
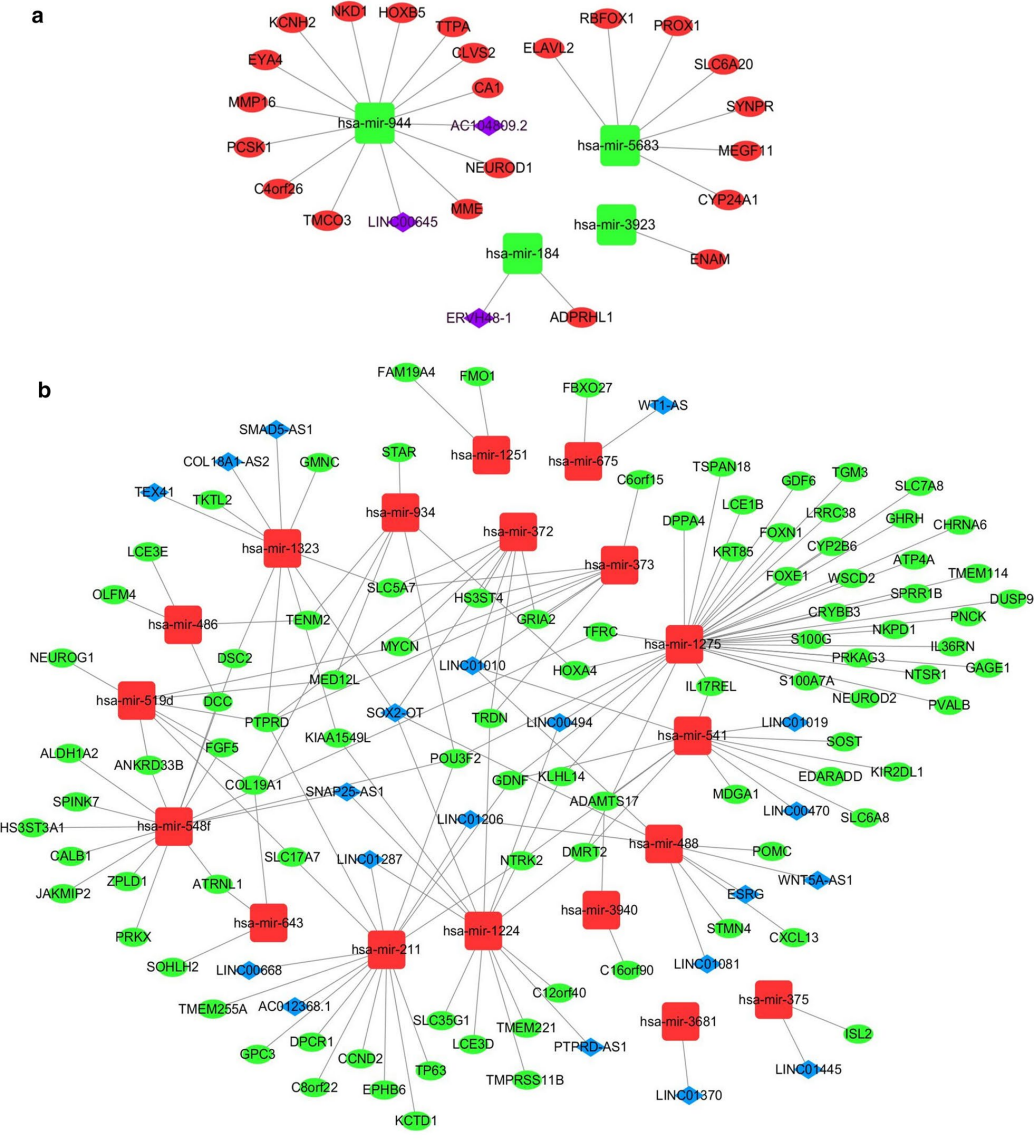
The metastasis-associated ceRNA network in lung cancer. **a** Green squares represent downregulated miRNAs, red ellipses represent upregulated mRNAs and purple diamonds represent upregulated lncRNAs. **b** Red squares represent upregulated miRNAs, green ellipses represent downregulated mRNAs and blue diamonds represent downregulated lncRNAs

Based on the above findings (Additional file 1: Tables S1, S2), we constructed the ceRNA network using Cytoscape 3.6. Figure 3 shows that 4 downregulated miRNAs, 22 upregulated mRNAs and 3 upregulated lncRNAs are involved in one ceRNA network. Meanwhile, 18 upregulated miRNAs, 95 downregulated mRNAs and 20 downregulated lncRNAs were involved in another ceRNA network.

### Identification of a prognostic signature of lung cancer

**Fig. 4:**
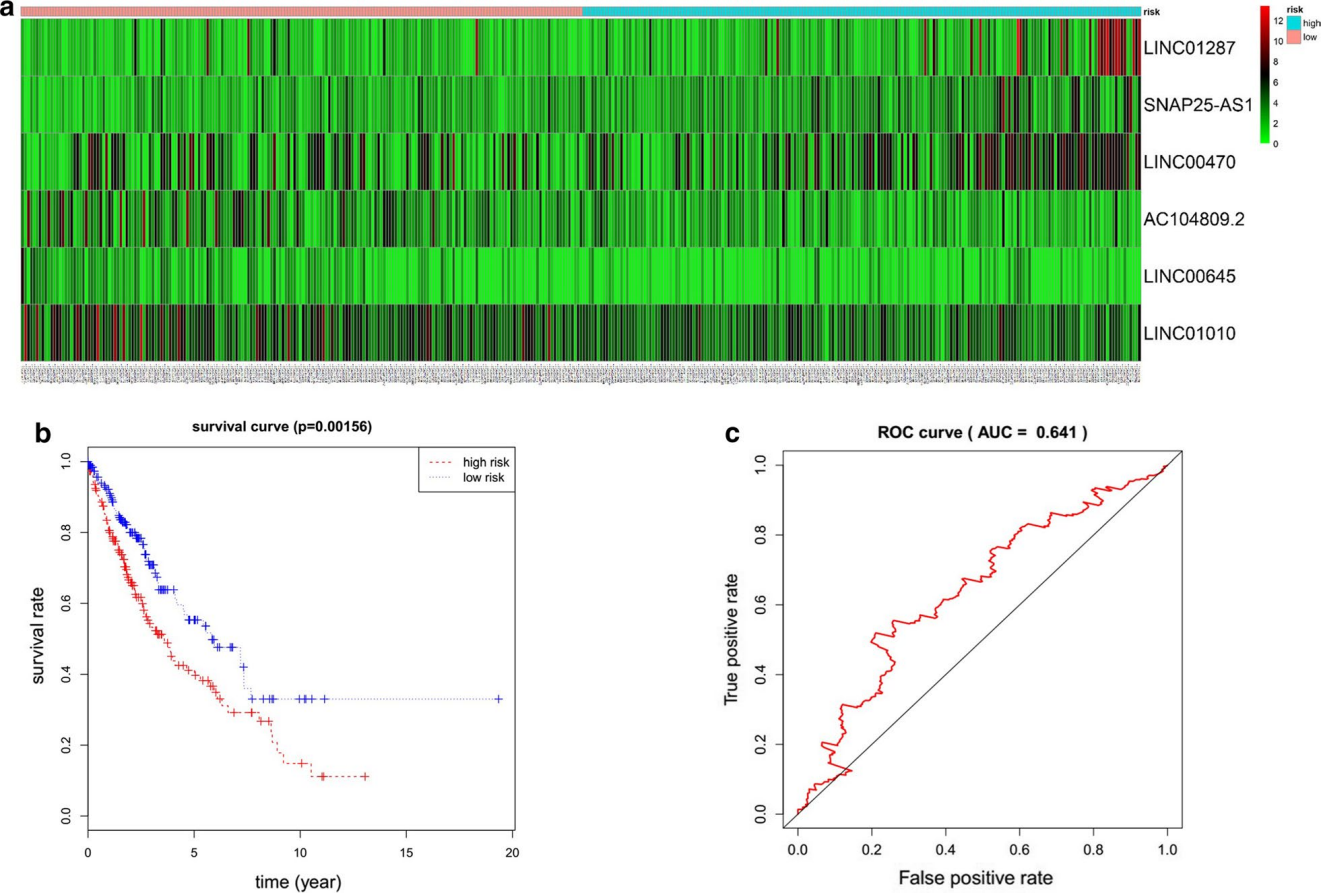
Evaluation of the 6 lncRNA signature in the training set. **a** Lung cancer patients in the training set were divided into high- and low-risk groups based on their risk scores generated from the 6 lncRNA signature. The expression heat map shows the expression profiles of the 6 lncRNAs in lung cancer patients. **b** Kaplan–Meier survival curve analysis for overall survival of lung cancer patients using the risk scores generated from the 6 lncRNA signature. Differences between high-risk (n = 194) and low-risk (n = 193) groups were determined by log-rank test (P = 0.016). **c** Validation of the 6 lncRNA signature by ROC curve for predicting 10-year survival in lung cancer patients based on risk scores

Increasing studies have shown that lncRNAs effectively predict overall survival (OS) in cancer patients [28, 29]. To validate the association between the lncRNAs in the ceRNA network and OS of lung cancer, we randomly divided lung cancer patients into the training set (n = 387) and testing set (n = 386), and there was no significant difference in the clinical covariates between the two sets (P > 0.05) (Additional file 1: Table S5). We analyzed the 23 DE lncRNAs in the training set by multivariate Cox regression analysis. The results demonstrated that a 6 lncRNA signal was a significant prognostic factor for lung cancer (Additional file 1: Table S6). LINC01287, SNAP25-AS1 and LINC00470 were protective, whereas AC104809.2, LINC00645 and LINC01010 were associated with increased risk (Fig. 4a). Next, lung cancer patients in the training set were divided into high‐risk and low‐risk groups based on the following risk score formula:

$$ \begin{aligned} {\text{Risk Score}} &= \left( {0.0 6 6 2} \right) \, *{\text{LINC}}0 1 2 8 7+ \, \left( {0.0 9 7 8} \right) \, *{\text{ SNAP25}} \hfill \\ & \quad- {\text{AS1}} + \, \left( {0.0 5 6 7} \right) \, *{\text{LINC}}00 4 70 + \, \left( { - 0.0 7 4 5} \right) \, *{\text{AC1}}0 4 80 9. 2\hfill \\ & \quad + \, \left( { - 0. 20 1 8} \right) \, *{\text{LINC}}00 6 4 5+ \, \left( { - 0.0 7 2 6} \right) \, *{\text{LINC}}0 10 10. \hfill \\ \end{aligned} $$

Kaplan–Meier analysis revealed that OS in the high-risk group was significantly lower than in the low-risk group (*P *= 0.0016, Fig. 4b). The overall 10-year relative survival rates of the high-risk and the low-risk groups were 14.9% and 33%, respectively. Furthermore, the area under ROC curve (AUC) at 10 years for OS was 0.641 (Fig. 4c).

**Fig. 5:**
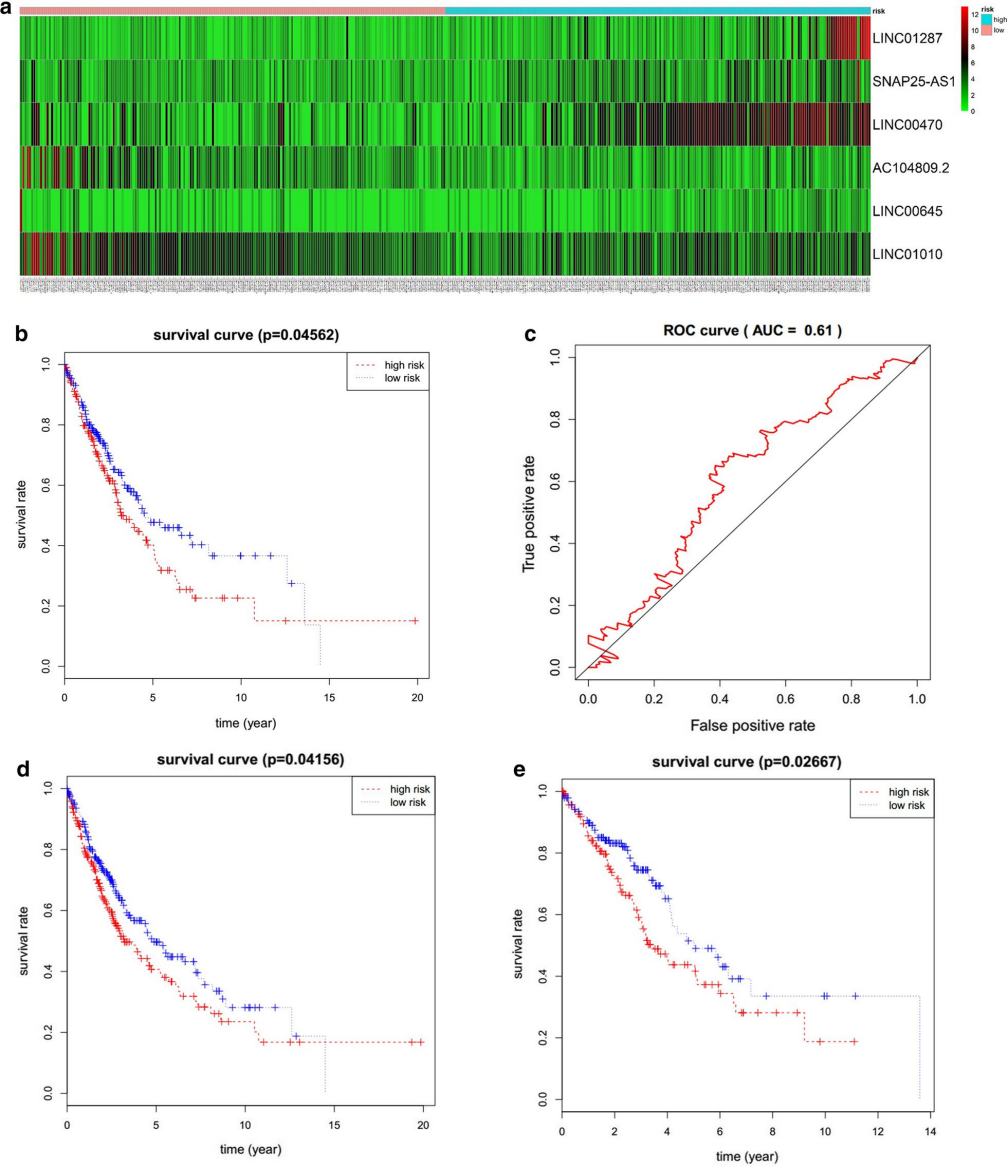
Prognostic evaluation of the 6 lncRNA signature in the testing set and stratification analysis by gender. **a** Lung cancer patients in the testing set were divided into high- and low-risk groups based on their risk scores generated from the 6 lncRNA signature. The expression heat map shows the expression profiles of the 6 lncRNAs in lung cancer patients. **b** Kaplan–Meier survival curve analysis for overall survival of lung cancer patients using the risk scores generated from the 6 lncRNA signature. Differences between high-risk (n = 193) and low-risk (n = 193) groups were determined by log-rank test (P = 0.046). **c** Validation of the 6 lncRNA signature by ROC curve for predicting 10-year survival in lung cancer patients based on risk scores; Kaplan–Meier survival curve analysis of overall survival in high-risk and low-risk groups for male patients **d** and female patients **e**

In order to validate the prognostic ability of the 6-lncRNA model, we were further performed Kaplan–Meier analysis and ROC curve analysis in the testing set. Lung cancer patients in the testing set were divided into high-risk and low-risk group (Fig. 5a) with statistically significant different overall survival (*P *= 0.046, Fig. 5b) and ROC curve (AUC = 0.61, Fig. 5c).

Next, all lung cancer patients were stratified by gender. The six-lncRNA signature could classify 486 male patients into high-risk group and low-risk group and there was the statistically significant difference between the high-risk and the low-risk group in Kaplan–Meier analysis (P = 0.042, Fig. 5d). Similarly, 287 female patients were divided into high-risk group and low-risk group with statistically significant different overall survival (P = 0.027, Fig. 5e). These results indicate that the 6 lncRNA signal is indeed an independent prognostic factor for predicting OS in lung cancer patients.

### Clinical feature analysis of LINC01010

**Fig. 6:**
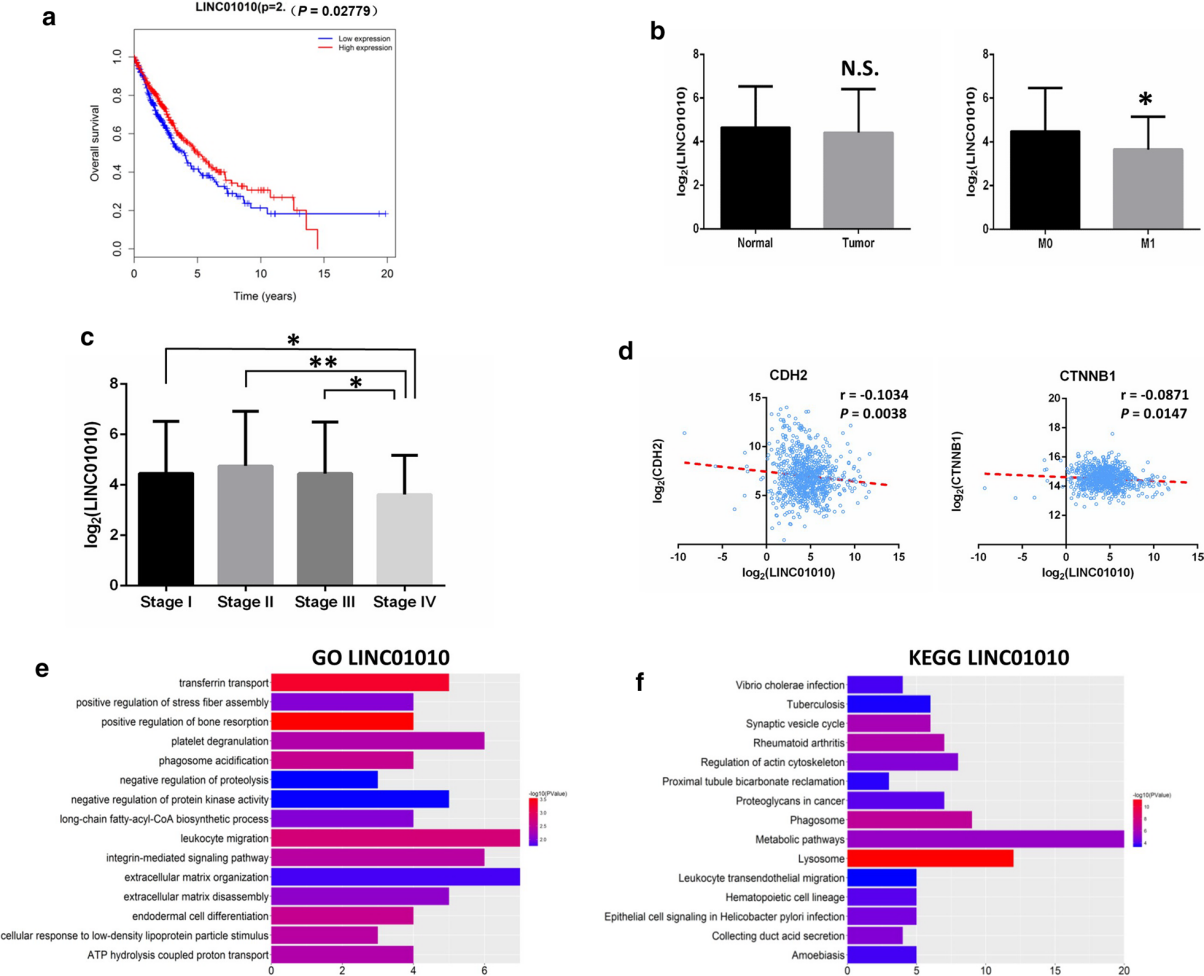
Clinical feature analysis of LINC01010. **a** Kaplan–Meier analysis of lung cancer overall survival according to expression of LINC01010. **b** LINC01010 expression analysis in lung cancer tissues and normal tissues (left). LINC01010 expression analysis in metastatic lung cancer tissues (M1) and nonmetastatic tissues (M0) (right). **c** LINC01010 expression analysis in stage I–IV lung cancer. **d** LINC01010 was negatively correlated with EMT markers (CDH2 and CTNNB1) in lung cancer. **e**, **f** Top 15 GO and KEGG pathways of genes co-expressed with LINC01010. Color from blue to red indicates − log10(*P*-value) from low to high. X-axes represent the number of genes involved in each pathway. N.S. not significant, *P < 0.05, **P < 0.01

To further analyze whether these 6 lncRNAs affect clinical features in lung cancer from TCGA database, we divided lung cancer patients into high-expression and low-expression groups based on lncRNA expression. Kaplan–Meier survival curves revealed that only LINC01010 was positively correlated with OS (P = 0.02779) (Fig. 6a). OS at 5 and 10 years in the LINC01010 high-expression group was 49.9% and 26.8%, respectively, compared with 40.6% and 18.2% in the low-expression group. Therefore, LINC01010 was chosen for further study. Although there was no significant difference between normal tissues and lung cancer tissues, expression levels of LINC01010 were downregulated in M1 (metastatic samples) (Fig. 6b) and in stage IV tumors (Fig. 6c) compared to M0 (nonmetastatic samples) and stage I, respectively. We further investigated whether LINC01010 was related to epithelial–mesenchymal transition (EMT) markers, which can be used to indicate an increased capacity for migration of tumor cells [26, 30]. Pearson correlation analysis showed that LINC01010 was negatively correlated with EMT markers (CDH2 and CTNNB1) in lung cancer patient samples (Fig. 6d).

To further investigate the function of LINC01010, we performed co-expression analysis of LINC01010 using the Multi-Experiment Matrix (MEM) resource and selected the top 200 mRNAs co-expressed with LINC01010 for GO and KEGG pathway enrichment analysis (Additional file 1: Table S7). The top 15 biological processes identified in GO and KEGG pathways revealed that mRNAs positively co-expressed with LINC01010 participated primarily in “integrin-mediated signaling pathway”, “extracellular matrix organization”, “leukocyte migration”, “regulation of actin cytoskeleton” and “proteoglycans in cancer” (Fig. 6e, f), suggesting that LINC01010 may regulate tumorigenesis and metastasis in lung cancer [31, 32].

### LINC01010 represses lung cancer cell migration, but not proliferation

**Fig. 7:**
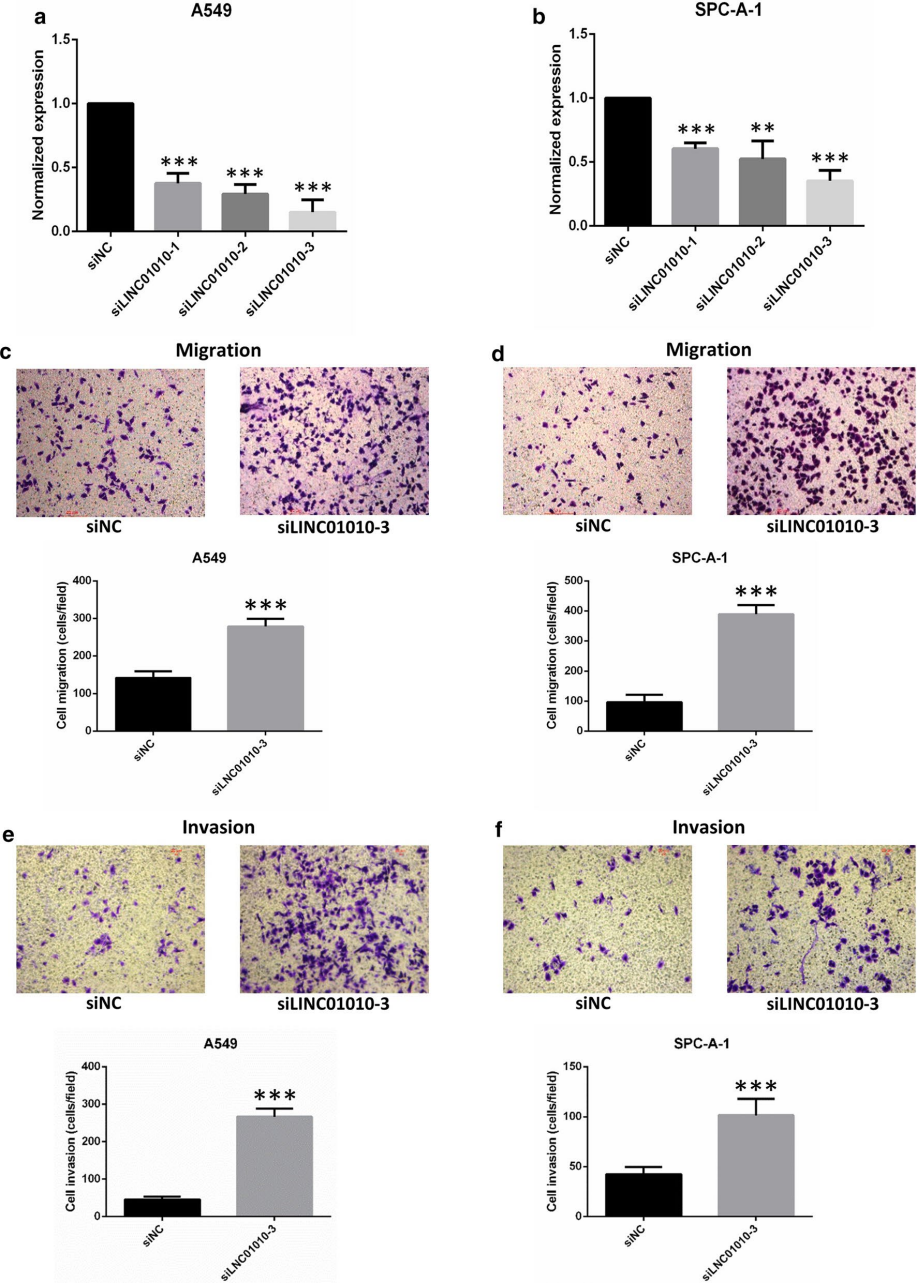
LINC01010 represses lung cancer cell migration. LINC01010 expression levels were determined in siNC and siLINC01010 transfected A549 **a** or SPC-A-1 **b** cells by qRT-PCR. Expression levels of LINC01010 were normalized to GAPDH. Transwell assays were used to determine the migration and invasive abilities of siNC and siLINC01010 transfected A549 **c**, **e** and SPC-A-1 **d**, **f** cells. All data are represented as the mean ± SD (n = 3), **P< 0.01, ***P < 0.001

To further determine whether LINC01010 affects lung cancer cell migration ability, we knocked down its expression by transfecting siRNA for LINC01010 into A549 or SPC-A-1 cells. qRT-PCR results showed that expression levels of LINC01010 were significantly decreased in three siRNA-transfected cells compared with controls (Fig. 7a, b). SiLINC01010-3 was selected for subsequent functional experiments. Transwell assays showed that suppression of LINC01010 promoted both A549 (Fig. 7c, e) and SPC-A-1 cell (Fig. 7d, f) migration and invasion compared with control cells, which were also consistent with the negatively correlation with EMT markers (Fig. 6d). However, either in A549 cells or SPC-A-1 cells, transfection of LINC01010 siRNA had little effect on the proliferation of lung cancer cells (Additional file 2: Fig. S1). In sum, LINC01010 inhibits the migratory and invasive capacity of lung cancer cells, but not proliferation.

### LINC01010 associated ceRNA network

**Fig. 8:**
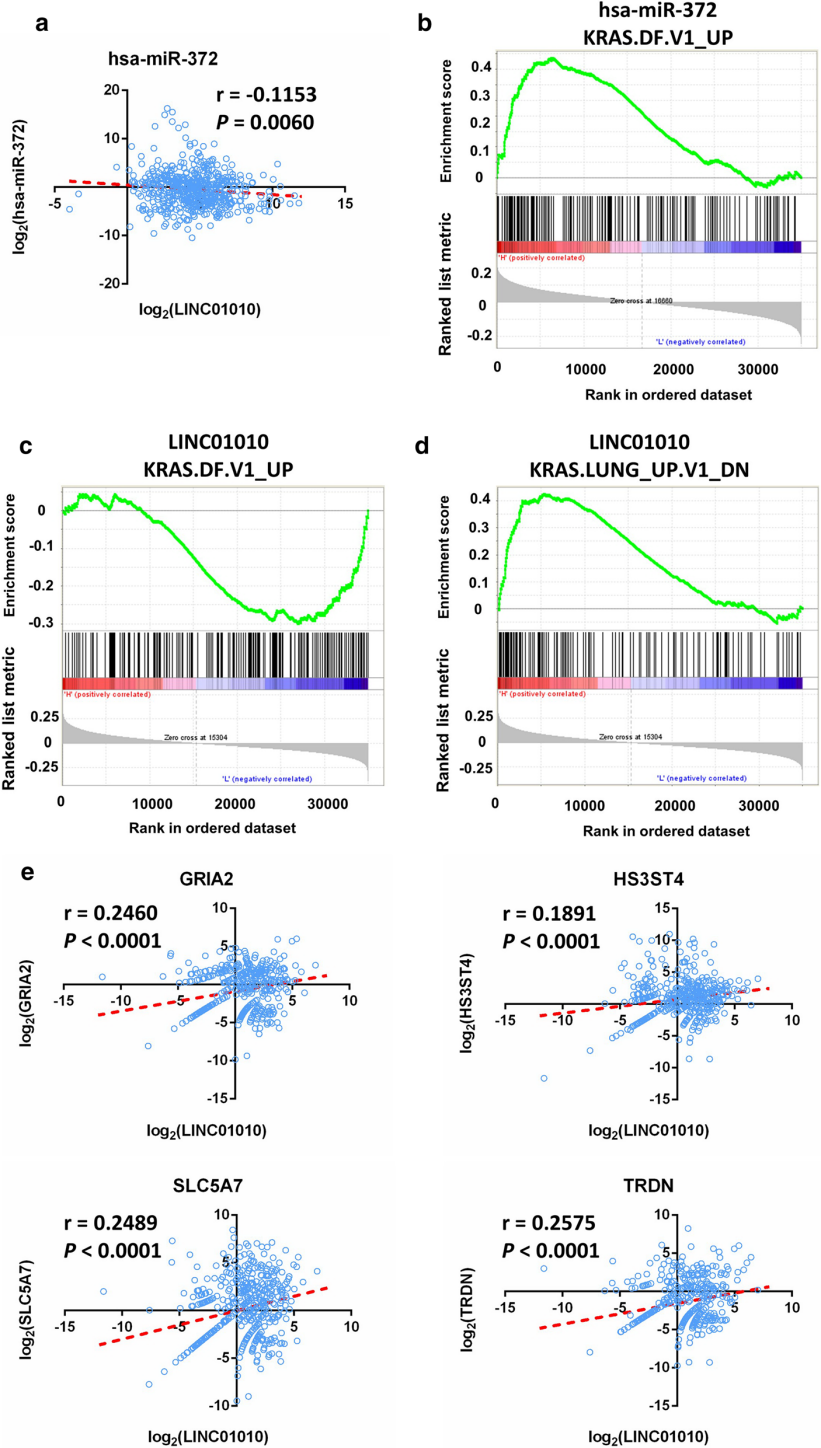
LINC01010 is negatively correlated with miR-372. **a** Pearson correlation analysis shows that LINC01010 is negatively correlated with hsa-mir-372 (r = − 0.1153, *P* = 0.0060). **b** GSEA results showed a correlation between hsa-mir-372 levels and the MAPK signaling pathway in lung cancer. The gene set “KRAS.DF.V1_UP” (*P* < 0.0001) was significantly enriched in high levels of hsa-mir-372. **c** The gene set “KRAS.DF.V1_UP” (*P* = 0.011) was significantly enriched in low levels of LINC01010. **d** “KRAS.LUNG_UP.V1_DN” (*P* < 0.0001) was significantly enriched in high levels of LINC01010. **e** Pearson correlation analysis showed that LINC01010 is positively correlated with GRIA2 (r = 0.246, *P* < 0.0001), HS3ST4 (r = 0.1891, *P* < 0.0001), SLC5A7 (r = 0.2489, *P* < 0.0001) and TRDN (r = 0.2575, *P* < 0.0001)

The ceRNA networks illustrated that LINC01010 may target hsa-mir-372, hsa-mir-373, hsa-mir-488 and hsa-mir-541. By Pearson correlation analysis, we found that LINC01010 was negatively correlated with hsa-mir-372 (Fig. 8a). By GSEA analysis, the gene set “KRAS.DF.V1_UP” (*P* < 0.0001) was significantly enriched in high levels of hsa-mir-372 (Fig. 8b), which was also verified by the literature [33, 34]. Meanwhile, the gene set “KRAS.DF.V1_UP” (*P* = 0.011) was significantly enriched in low levels of LINC01010 (Fig. 8c) and “KRAS.LUNG_UP.V1_DN” (*P* < 0.0001) was significantly enriched in high levels of LINC01010 (Fig. 8d), which suggested that LINC01010 might inhibit the function of hsa-mir-372 in the MAPK pathway.

We further investigated whether expression of LINC01010 was positively correlated with hsa-mir-372 target mRNAs. Pearson correlation analysis showed that LINC01010 was positively correlated with GRIA2, HS3ST4, SLC5A7 and TRDN (Fig. 8e). Therefore, LINC01010 may play an important role in tumorigenesis in lung cancer by competing with miR-372.

## Discussion

Lung cancer is the most prevalent cancer with the highest incidence and mortality rates [1]. Since most patients present with invasive tumors, it is crucial to understand the molecular mechanisms of lung cancer metastasis. Increasingly, studies have shown that lncRNAs play important roles in the pathogenesis of lung cancer [3–6], which may act as ceRNAs to regulate mRNAs expression [16]. A recent study constructed a ceRNA network between lung adenocarcinoma samples and adjacent nontumor lung tissues samples [35]. However, the role of ceRNA in metastatic lung cancer remains unclear.

In the present study, we constructed a lncRNAs-miRNAs-mRNAs ceRNA network between metastatic lung cancer and nonmetastatic lung cancer patients based on TCGA database. We then divided DE mRNAs into upregulated and downregulated groups and performed GO and KEGG analysis on these DE mRNAs to further investigate pathways involved in metastatic lung cancer. In the upregulated group, GO:0003341 ~ cilium movement and “MAPK signaling pathway” were observed, which have been reported to be associated with lung cancer. Numerous lung diseases are marked by abnormalities in both cilia structure and function [36]. The primary cilium provides a spatially localized platform for signaling by Hedgehog, Notch and WNT, which promotes metastasis in lung cancer [24]. Furthermore, the MAPK pathway is activated in non–small cell lung tumors and plays a key role in migration and invasion of lung cancer [25, 37]. While downregulated mRNAs participated in “GO:0008544 ~ epidermis development” and “drug metabolism-cytochrome P450”. EMT is considered an essential process for metastasis [26, 30]. During this process, epithelial markers, such as keratins, which are primarily clustered in “GO:0008544 ~ epidermis development”, are downregulated [27]. Several studies have shown that glutathione S-transferase (GST) polymorphisms increase the risk for various cancers, including lung cancer [38]. Moreover, low expression of GSTM3, which is involved in “drug metabolism-cytochrome P450”, correlates with increased susceptibility to lung cancer [39, 40].

To our knowledge, few articles have identified lung cancer-specific lncRNAs as molecular biomarkers for diagnosis and prognosis. Therefore, we performed multivariate Cox regression analysis to analyze the 23 metastasis-associated DE lncRNAs. Result revealed a 6 lncRNA signal, including LINC01287, SNAP25-AS1, LINC00470, AC104809.2, LINC00645 and LINC01010, as an independent prognostic factor for predicting OS in lung cancer patients. According to the network topology analysis, the degree distribution density map of nodes indicated that most nodes in the ceRNA network are isolated (Additional file 3: Fig. S2). Among the 6 lncRNA signature, the node degrees of SNAP25-AS1, LINC01287 and LINC01010 are 2, 2, 4, respectively. The closeness centrality of the 6 lncRNA signature exceeds most lncRNAs in the ceRNA network. Therefore, the 6 lncRNA signature might be the key nodes that affect neighboring nodes and act as a biomarker for lung cancer metastasis.

Furthermore, we noted that expression of LINC01010 was positively correlated with OS and negatively correlated with metastasis and lung cancer stage. Therefore, we postulate that LINC01010 may play a more crucial role in the pathogenesis and prognosis of lung cancer. GO and KEGG pathway analyses demonstrated that mRNAs positively co-expressed with LINC01010 participate in “integrin-mediated signaling pathway”, “extracellular matrix organization”, “leukocyte migration”, “regulation of actin cytoskeleton” and “proteoglycans in cancer”, which reportedly participate in tumorigenesis and metastasis of lung cancer [31, 32]. These in silico analysis results were confirmed by our functional experiments. Transwell assays showed that suppression of LINC01010 inhibited lung cancer cell migration and invasion.

Dysregulated miRNA expression is also reported to play crucial roles in the carcinogenesis of lung cancer [41]. In the present study, the ceRNA networks we constructed illustrate that LINC01010 may target hsa-mir-373, hsa-mir-488, hsa-mir-541, and hsa-mir-372. Huang et al. demonstrated that miR-373 stimulates cancer cell migration and invasion in vitro and in vivo and that certain cancer cell lines depend on endogenous miR-373 activity to efficiently migrate [42]. Voorhoeve et al. indicated that either miR-373 or miR-372 prevents RAS-induced growth arrest in primary human cells, possibly through inhibition of expression of the tumor suppressor LATS2 [43]. Moreover, hsa-mir-372 promotes invasive ability of lung cancer cells and globally affects the regulatory circuits centered on MAPK/ERK signaling [33, 34]. By Pearson correlation analysis, we found that expression of LINC01010 negatively correlated with hsa-mir-372 and positively correlated with hsa-mir-372 target genes. By GSEA analysis, we found that LINC01010 might inhibit the function of hsa-mir-372 in the MAPK pathway (Fig. 8b–d). In addition, we found that LINC01010 was positively correlated with the P53 pathway (*P* < 0.0001, Additional file 4: Fig. S3a), while hsa-mir-372 and the P53 pathway showed a negative correlation trend (P = 0.17, Additional file 4: Fig. S3c). Meanwhile, hsa-mir-372 was positively correlated with the TGFβ signaling pathway (P = 0.037, Additional file 4: Fig. S3d). The gene set “TGF_BETA_SIGNALING” was enriched in low levels of LINC01010 but no statistical difference (P = 0.12, Additional file 4: Fig. S3b). These results not only indicated that LINC01010 was a tumor suppressor, but also confirmed that LINC01010 involved in lung cancer ceRNA by competing with hsa-mir-372. To gain further insight into the potential role of LINC01010, we will investigate whether LINC01010 sponges hsa-mir-372 by binding to its response elements in the future.

## Conclusions

We constructed a metastasis-associated ceRNA network in lung cancer and identified a 6 lncRNA signature that is an independent prognostic factor for predicting OS in lung cancer. For the first time, we validated LINC01010 as a tumor suppressor lncRNA in lung cancer through bioinformatics and Transwell assay experiments. Our findings provide insight into the identification of potential lncRNA biomarkers for diagnosis and prognosis in lung cancer.

## Supplementary information

**Additional file 1.** Supplementary tables.

**Additional file 2: Fig. S1.** LINC01010 dose not affect the proliferation of lung cancer cells. CCK8 assays were used to assess the role of LINC01010 siRNA in the proliferation of A549 cells (**a**) or SPC-A-1 cells (**b**).

**Additional file 3: Fig. S2.** Topology analysis of ceRNA network. **a** The node degree distribution shows that the nodes with less response are the majority. **b** The closeness centrality of many nodes in the ceRNA network are relatively concentrated and similar. **c** The shortest path length distribution of the ceRNA network is scattered. **d** The degree distribution density map of nodes indicates that most nodes in the ceRNA network are isolated.

**Additional file 4: Fig. S3.** LINC01010 and hsa-mir-372 affect the P53 pathway and the TGFβ signaling pathway. **a** The gene set “HALLMARK_P53_PATHWAY” was significantly enriched in high levels of LINC01010 (P < 0.0001). **b** The gene set “ HALLMARK_TGF_BETA_SIGNALING” was enriched in low levels of LINC01010 but no statistical difference (P = 0.12). **c** hsa-mir-372 and P53 pathway showed a negative correlation trend without statistical difference (P = 0.17). **d** The gene set “HALLMARK_TGF_BETA_SIGNALING” was significantly enriched in high levels of hsa-mir-372 (P = 0.037).

## Data Availability

Authors can provide all of datasets analyzed during the study on reasonable request.

## Abbreviations

mRNA: Messenger RNA
miRNA: MicroRNA
lncRNA: Long non-coding RNA
ceRNAs: Competing endogenous RNAs
TCGA: The genomic data commons data portal and the cancer genome atlas database
GDC: The genomic data commons data portal
GO: Gene ontology database
KEGG: Kyoto encyclopedia of genes and genomes
MEM: The multi-experiment matrix resource
DAVID: Database for annotation, visualization, and integrated discovery
KOBAS: KEGG orthology based annotation system
GSEA: Gene set enrichment analysis
OS: Overall survival
AIC: Akaike Information Criterion
ROC curve: Receiver operating characteristic curve
AUC: Area under curve
DE: Differentially expressed
FDR: False discovery rate
siLNC01010: A small interfering RNA for LNC01010
siRNA‐NC: A negative control
