# Supplementary material for: Construction of a metastasis-associated ceRNA network reveals a prognostic signature in lung cancer
Source: Cancer Cell Int. 2020 Jun 3;20:208. doi: 10.1186/s12935-020-01295-8 (PMC7271455; doi:10.1186/s12935-020-01295-8)
Supplement: Supplementary file 1 — Additional file 1. Supplementary tables. [file 12935_2020_1295_MOESM1_ESM.docx]

**TABLE S1** MiRNAs targeting metastasis lung cancer specific mRNAs

| **miRNAs** | **mRNAs** |
| --- | --- |
| hsa-mir-3923 | ENAM |
| hsa-mir-5683 | MEGF11, ELAVL2, CYP24A1, RBFOX1, SLC6A20, SYNPR, PROX1 |
| hsa-mir-184 | ADPRHL1 |
| hsa-mir-944 | MMP16, PCSK1, NKD1, CA1, MME, TTPA, NEUROD1, EYA4, HOXB5, C4orf26, TMCO3, KCNH2, CLVS2 |
| hsa-mir-1224 | TRDN, TMEM221, DMRT2, C12orf40, LCE3D, KIAA1549L, KLHL14, SLC35G1, TMPRSS11B |
| hsa-mir-1251 | FMO1, FAM19A4 |
| hsa-mir-1275 | KLHL14, PVALB, CHRNA6, GHRH, GAGE1, SPRR1B, DUSP9, GDF6, IL17REL, POU3F2, CYP2B6, FOXN1, DMRT2, TGM3, LCE1B, CRYBB3, NEUROD2, GDNF, DPPA4, S100G, SLC7A8, TMEM114, LRRC38, WSCD2, TFRC, KRT85, S100A7A, HOXA4, TSPAN18, IL36RN, ATP4A, PRKAG3, NTSR1, PNCK, NKPD1, FOXE1 |
| hsa-mir-1323 | DSC2, PTPRD, SLC5A7, KIAA1549L, GMNC, TKTL2 |
| hsa-mir-211 | NTRK2, SLC17A7, CCND2, KCTD1, EPHB6, GPC3, POU3F2, C8orf22, TP63, GDNF, DPCR1, TMEM255A, PTPRD |
| hsa-mir-372 | MYCN, HS3ST4, SLC5A7, GRIA2, PTPRD, TRDN |
| hsa-mir-373 | HS3ST4, C6orf15, TRDN, GRIA2, SLC5A7, MYCN, PTPRD |
| hsa-mir-375 | ISL2 |
| hsa-mir-3940 | C16orf90, ADAMTS17 |
| hsa-mir-486 | DCC, OLFM4, TENM2, LCE3E |
| hsa-mir-488 | STMN4, POMC, CXCL13 |
| hsa-mir-519d | FGF5, NEUROG1, PTPRD, COL19A1, ANKRD33B, MYCN, MED12L, SLC17A7 |
| hsa-mir-541 | SOST, IL17REL, SLC6A8, GDNF, MDGA1, DMRT2, KIR2DL1, EDARADD, NTRK2, ADAMTS17 |
| hsa-mir-548f | TFRC, JAKMIP2, PRKX, SPINK7, CALB1, ATRNL1, DCC, FGF5, ANKRD33B, DSC2, ZPLD1, ALDH1A2, HS3ST3A1, POU3F2 |
| hsa-mir-643 | ATRNL1, SOHLH2, COL19A1 |
| hsa-mir-675 | FBXO27 |
| hsa-mir-934 | TENM2, STAR, MED12L, COL19A1, HOXA4, POU3F2 |

**TABLE S2** MiRNAs targeting metastasis lung cancer specific lncRNAs

| **miRNAs** | **lncRNAs** |
| --- | --- |
| hsa-mir-184 | ERVH48-1 |
| hsa-mir-944 | LINC00645, AC104809.2 |
| hsa-mir-1224 | LINC00494, SNAP25-AS1, PTPRD-AS1, LINC01287, SOX2-OT |
| hsa-mir-1275 | LINC00494 |
| hsa-mir-1323 | SMAD5-AS1, SOX2-OT, COL18A1-AS2, TEX41 |
| hsa-mir-211 | LINC01206, AC012368.1, LINC00668, LINC01287 |
| hsa-mir-3681 | LINC01370 |
| hsa-mir-372 | LINC01010, SOX2-OT |
| hsa-mir-373 | LINC01010 |
| hsa-mir-375 | LINC01445 |
| hsa-mir-488 | ESRG, LINC01010, SOX2-OT, WNT5A-AS1, LINC01081, LINC01206 |
| hsa-mir-541 | LINC00470, LINC01019, LINC01010 |
| hsa-mir-548f | SNAP25-AS1 |
| hsa-mir-675 | WT1-AS |

**TABLE S3** Metastasis lung cancer specific lncRNAs in ceRNA network construction

| **lncRNAs** | **Ensembl ID** | **Regulation** | **logFC** | **PValue** | **FDR** |
| --- | --- | --- | --- | --- | --- |
| ESRG | ENSG00000265992 | Downregulation | -7.07006 | 1.71E-06 | 0.000198 |
| LINC01370 | ENSG00000237767 | Downregulation | -6.88727 | 1.09E-05 | 0.0009 |
| LINC01287 | ENSG00000234722 | Downregulation | -4.77952 | 9.01E-07 | 0.000111 |
| LINC01206 | ENSG00000242512 | Downregulation | -4.51019 | 2.47E-05 | 0.001767 |
| LINC01445 | ENSG00000231427 | Downregulation | -3.89969 | 0.003104 | 0.048483 |
| LINC01019 | ENSG00000248118 | Downregulation | -3.7594 | 0.000504 | 0.015229 |
| LINC00668 | ENSG00000265933 | Downregulation | -2.28304 | 0.001413 | 0.029182 |
| LINC00470 | ENSG00000132204 | Downregulation | -2.13617 | 0.000649 | 0.017438 |
| LINC01081 | ENSG00000268754 | Downregulation | -2.1059 | 0.001642 | 0.03222 |
| LINC01010 | ENSG00000236700 | Downregulation | -1.98877 | 1.19E-05 | 0.000978 |
| SOX2-OT | ENSG00000242808 | Downregulation | -1.92725 | 0.000762 | 0.019449 |
| COL18A1-AS2 | ENSG00000224574 | Downregulation | -1.8597 | 0.000389 | 0.012723 |
| LINC00494 | ENSG00000235621 | Downregulation | -1.6795 | 3.95E-05 | 0.002511 |
| WT1-AS | ENSG00000183242 | Downregulation | -1.51794 | 0.000563 | 0.016211 |
| SNAP25-AS1 | ENSG00000227906 | Downregulation | -1.50051 | 0.001356 | 0.028481 |
| SMAD5-AS1 | ENSG00000164621 | Downregulation | -1.48845 | 2.52E-05 | 0.00178 |
| PTPRD-AS1 | ENSG00000225706 | Downregulation | -1.35634 | 0.000567 | 0.016211 |
| WNT5A-AS1 | ENSG00000244586 | Downregulation | -1.30278 | 0.00022 | 0.008856 |
| TEX41 | ENSG00000226674 | Downregulation | -1.17052 | 0.000642 | 0.017416 |
| AC012368.1 | ENSG00000225889 | Downregulation | -1.08505 | 0.00015 | 0.006638 |
| AC104809.2 | ENSG00000233392 | Upregulation | 1.941236 | 1.03E-07 | 1.58E-05 |
| LINC00645 | ENSG00000258548 | Upregulation | 3.38819 | 2.78E-25 | 2.79E-22 |
| ERVH48-1 | ENSG00000233056 | Upregulation | 3.432237 | 4.74E-21 | 3.29E-18 |

**TABLE S4** Metastasis lung cancer specific miRNAs in ceRNA network construction

| **miRNAs** | **Regulation** | **logFC** | **PValue** | **FDR** |
| --- | --- | --- | --- | --- |
| hsa-mir-3923 | Downregulation | -5.60113 | 4.45E-05 | 0.00217 |
| hsa-mir-5683 | Downregulation | -2.62932 | 0.000193 | 0.007473 |
| hsa-mir-184 | Downregulation | -2.38849 | 0.001403 | 0.025872 |
| hsa-mir-944 | Downregulation | -2.16097 | 0.000272 | 0.009126 |
| hsa-mir-372 | Upregulation | 4.139752 | 4.06E-13 | 2.78E-10 |
| hsa-mir-373 | Upregulation | 3.062021 | 3.61E-06 | 0.000206 |
| hsa-mir-3681 | Upregulation | 2.195191 | 7.54E-07 | 7.36E-05 |
| hsa-mir-1323 | Upregulation | 2.191444 | 0.002067 | 0.032833 |
| hsa-mir-1224 | Upregulation | 2.067581 | 2.61E-08 | 3.56E-06 |
| hsa-mir-375 | Upregulation | 2.048763 | 5.38E-10 | 1.22E-07 |
| hsa-mir-519d | Upregulation | 1.917477 | 0.002983 | 0.043348 |
| hsa-mir-548f | Upregulation | 1.916833 | 3.14E-06 | 0.000195 |
| hsa-mir-1251 | Upregulation | 1.820224 | 8.14E-05 | 0.003708 |
| hsa-mir-488 | Upregulation | 1.628983 | 0.000329 | 0.009763 |
| hsa-mir-541 | Upregulation | 1.47307 | 0.000208 | 0.007473 |
| hsa-mir-934 | Upregulation | 1.344305 | 0.000857 | 0.021346 |
| hsa-mir-675 | Upregulation | 1.340486 | 0.001311 | 0.024876 |
| hsa-mir-211 | Upregulation | 1.208208 | 0.002769 | 0.041108 |
| hsa-mir-1275 | Upregulation | 1.201445 | 0.000173 | 0.007386 |
| hsa-mir-3940 | Upregulation | 1.179236 | 1.01E-06 | 8.65E-05 |
| hsa-mir-643 | Upregulation | 1.144663 | 3.73E-09 | 6.37E-07 |
| hsa-mir-486 | Upregulation | 1.143206 | 1.47E-06 | 0.000101 |

**TABLE S5** Clinical covariates in the training and testing sets

| **Covariates** | **Group** | **Total**  **n=773** | **Training set**  **n=387** | **Testing set**  **n=386** | **P-value** |
| --- | --- | --- | --- | --- | --- |
| Survival time |  | 2.73 ± 0.09 | 2.76 ± 0.13 | 2.69 ± 0.13 | 0.674 |
| Vital status | Alive | 451 (58.3%) | 229 (59.2%) | 222 (57.5%) | 0.896 |
|  | Dead | 322 (41.7%) | 158 (40.8%) | 164 (42.5%) |  |
| Stage | I | 382 (50%) | 191 (49.8%) | 191 (50.1%) | 0.941 |
|  | II | 218 (28.5%) | 106 (27.7%) | 112 (29.4%) |  |
|  | III | 132 (17.3%) | 72 (18.8%) | 60 (15.8%) |  |
|  | IV | 32 (4.2%) | 14 (3.7%) | 18 (4.7%) |  |
| T stage | T1 | 199 (25.8%) | 102 (26.4%) | 97 (25.2%) | 0.976 |
|  | T2 | 449 (58.2%) | 228 (59.1%) | 221 (57.4%) |  |
|  | T3 | 86 (11.2%) | 39 (10.1%) | 47 (12.2%) |  |
|  | T4 | 37 (4.8%) | 17 (4.4%) | 20 (5.2%) |  |
| N stage | N0 | 482 (63.3%) | 237 (62.4%) | 245 (64.3%) | 0.584 |
|  | N1 | 184 (24.2%) | 88 (23.1%) | 96 (25.2%) |  |
|  | N2-3 | 95 (12.5%) | 55 (14.5%) | 40 (10.5%) |  |
| M stage | M0 | 741 (95.9%) | 373 (96.4%) | 368 (95.3%) | 0.766 |
|  | M1 | 32 (4.1%) | 14 (3.6%) | 18 (4.7%) |  |
| Age | <=65 | 330 (43.6%) | 171 (44.5%) | 159 (42.6%) | 0.870 |
|  | >65 | 427 (56.4%) | 213 (55.5%) | 214 (57.4%) |  |
| Gender | Female | 287 (37.1%) | 143 (37.0%) | 144 (37.3%) | 0.995 |
|  | Male | 486 (62.9%) | 244 (63.0%) | 242 (62.7%) |  |

**TABLE S6** Six lncRNA risk score model

| **LncRNAs** | **Coeffcient** | **Exp(coef)** | **Se(coef)** | **z** | **Multivariate p-value** |
| --- | --- | --- | --- | --- | --- |
| LINC01287 | 0.0662 | 1.0684 | 0.0330 | 2.00 | 0.045 |
| SNAP25-AS1 | 0.0978 | 1.1027 | 0.0493 | 1.98 | 0.048 |
| LINC00470 | 0.0567 | 1.0583 | 0.0305 | 1.86 | 0.063 |
| AC104809.2 | -0.0745 | 0.9282 | 0.0426 | -1.75 | 0.080 |
| LINC00645 | -0.2018 | 0.8173 | 0.0701 | -2.88 | 0.004 |
| LINC01010 | -0.0726 | 0.9300 | 0.0418 | -1.74 | 0.083 |

**TABLE S7** Top 200 mRNAs co-expressed with LINC01010 according to MEM

| **lncRNA** | **mRNAs** |
| --- | --- |
| LINC01010 | DCSTAMP, LIPA, SLC29A3, DOCK3, CD276, GM2A, MRAS, ITGAM, A2M, ITGAX, CSTB, SLC28A3, LIMK1, ATP6V1F, SLC38A6, SLC9A7, MFSD12, MATK, FABP5, CD52, ACOT7, RAP1GDS1, DAB2, SLC26A11, SPOCD1, CNIH3, ATP6V0D2, TRPV2, NRIP3, GLRX2, ACP5, FBP1, LPCAT2, TREM2, TNFSF14, RGCC, MMP12, NPC1, NEK6, MCOLN3, RAB7B, ADO, PI4K2A, GPC4, PDXK, SLC17A9, MREG, CD109, MOSPD1, SPP1, GLRX3P2, WDR41, FABP3, KCNJ1, SH3BGRL3, CYFIP1, RASAL2, ZNF804A, TM6SF1, MYO1E, ASPHD1, SDC2, NR1H3, DHRS11, DNAJC5B, NCAPH, BCAP31, ABHD2, SSR3, CA2, CTNS, ITGB2, MFSD7, PQLC2, PDLIM4, RASGRP3, CCND2, IGSF6, SLC1A4, UBASH3B, SLC12A6, CLN8, GLRX3, KCNN4, COL8A2, ACSL6, MYOF, CD9, CRABP2, SLC27A3, SBF2-AS1, CD1B, CD84, HMG20B, SPRY2, ST3GAL5, EPB41L2, CALU, TIMP3, MFHAS1, GALM, ITGB1BP1, HCST, CCDC88A, STX3, FDX1, ATP6V1H, SORT1, ABHD12, CKLF, MITF, FGR, GPNMB, ATP6V1A, COL6A1, LIMS1, PPA2, AHCYL1, PEA15, TMEM251 ,ZNF697 ,GALNT12, PLA2G7, FN1, CSF1, ABCG2 ,PDE3B, PBDC1, MDH1, ACOT13, TMEM38B, FLRT2, HPCAL1, SLAMF8, ANXA11, FAIM, AVPI1, CORO1C, ZMIZ1-AS1, HSD3B7, SLC39A11, HTT, KIAA0930, LY9, TBC1D1, TMEM91, ERMP1, RNU6-29P, VIM, PGBD5, MAFF, HYPK, YPEL4, FUCA2, PPT1, WWTR1, NCEH1, PMFBP1, GCLC, ATP1B1, CD63, NRP1, SLC17A5, CDH23, OTOA, IL1RN, PHLDA3, GSTO1, HEXB, ZFYVE16, ACTN1, RHEBP2, EVL, TMEM53, MIR1229, ATP9B, RTN4R, HTRA4, APOC4-APOC2, NRROS, TFRC, MAPK13, MSR1, METTL1, ALCAM, PEX19, APOE, COL22A1, ARL15, CCNH, REEP5, MMP9, SRD5A3, PTPRE, KBTBD8, CIAO1, OPN3, AP2S1, RNH1, SLAMF9 |
