# Supplementary figures and images for: Construction of a metastasis-associated ceRNA network reveals a prognostic signature in lung cancer
Source: Cancer Cell Int. 2020 Jun 3;20:208. doi: 10.1186/s12935-020-01295-8 (PMC7271455; doi:10.1186/s12935-020-01295-8)

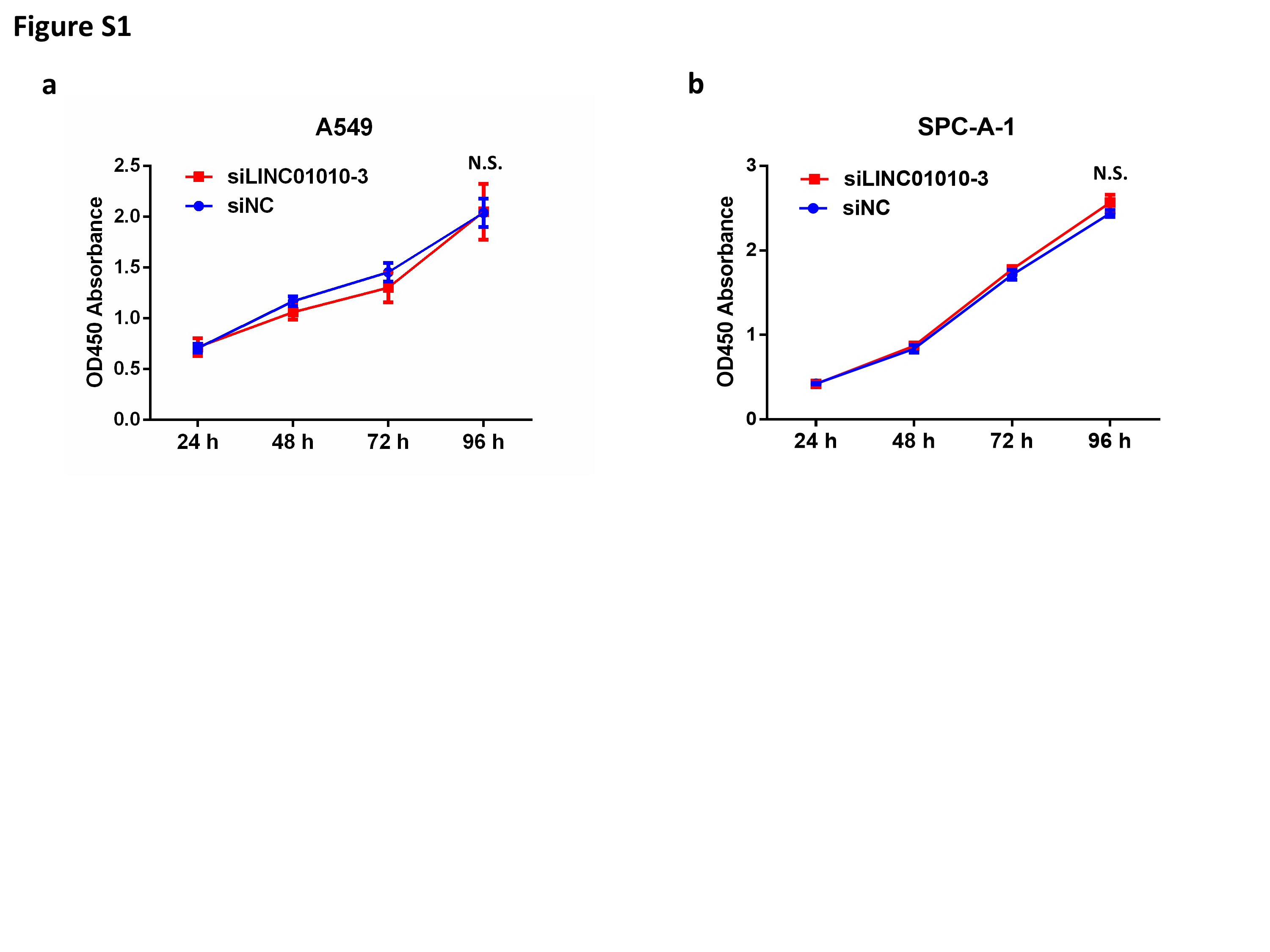

Supplement: Supplementary file 2 — Additional file 2: Fig. S1. LINC01010 dose not affect the proliferation of lung cancer cells. CCK8 assays were used to assess the role of LINC01010 siRNA in the proliferation of A549 cells (a) or SPC-A-1 cells (b). [file 12935_2020_1295_MOESM2_ESM.tif]

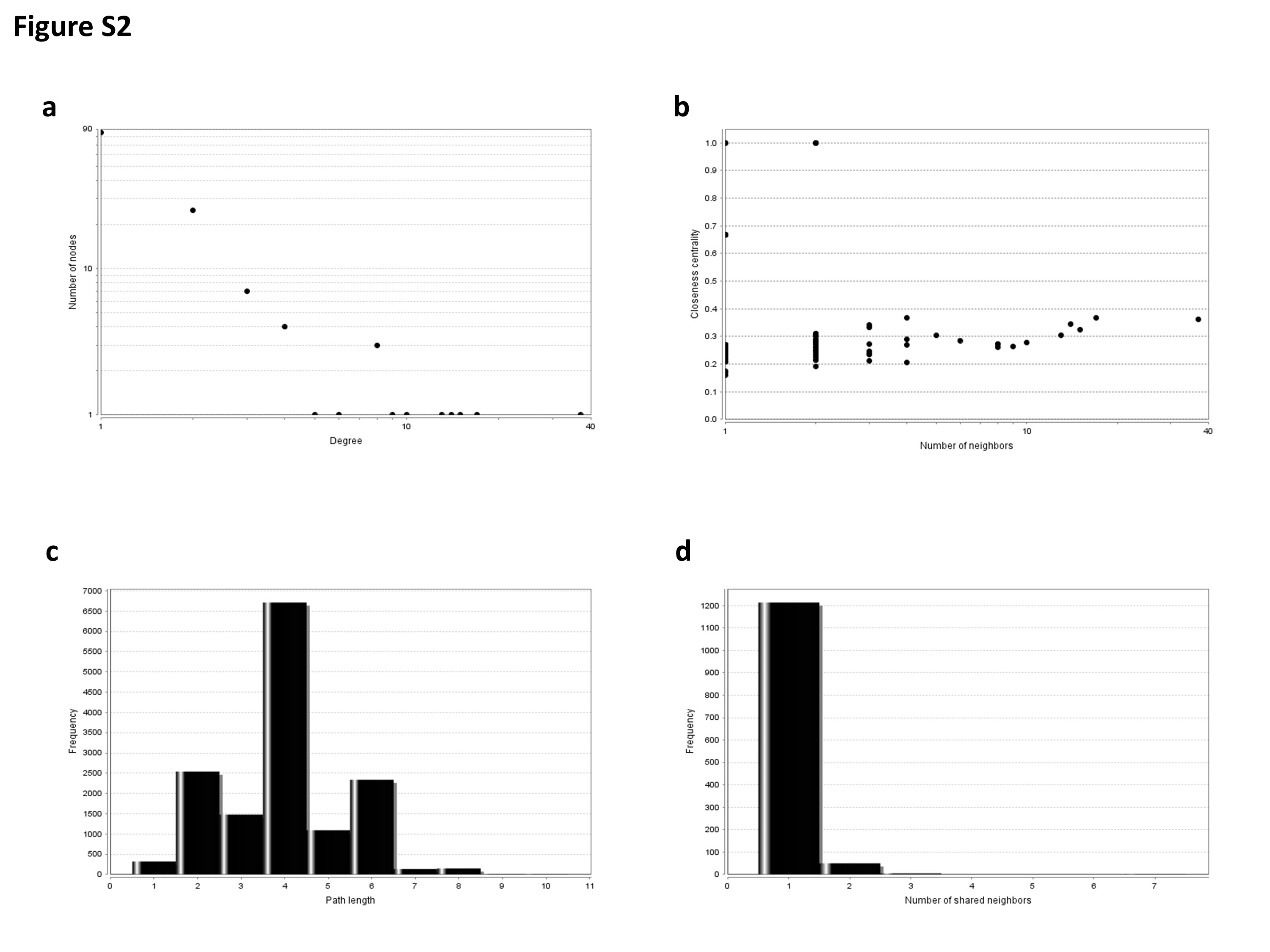

Supplement: Supplementary file 3 — Additional file 3: Fig. S2. Topology analysis of ceRNA network. a The node degree distribution shows that the nodes with less response are the majority. b The closeness centrality of many nodes in the ceRNA network are relatively concentrated and similar. c The shortest path length distribution of the ceRNA network is scattered. d The degree distribution density map of nodes indicates that most nodes in the ceRNA network are isolated. [file 12935_2020_1295_MOESM3_ESM.tif]

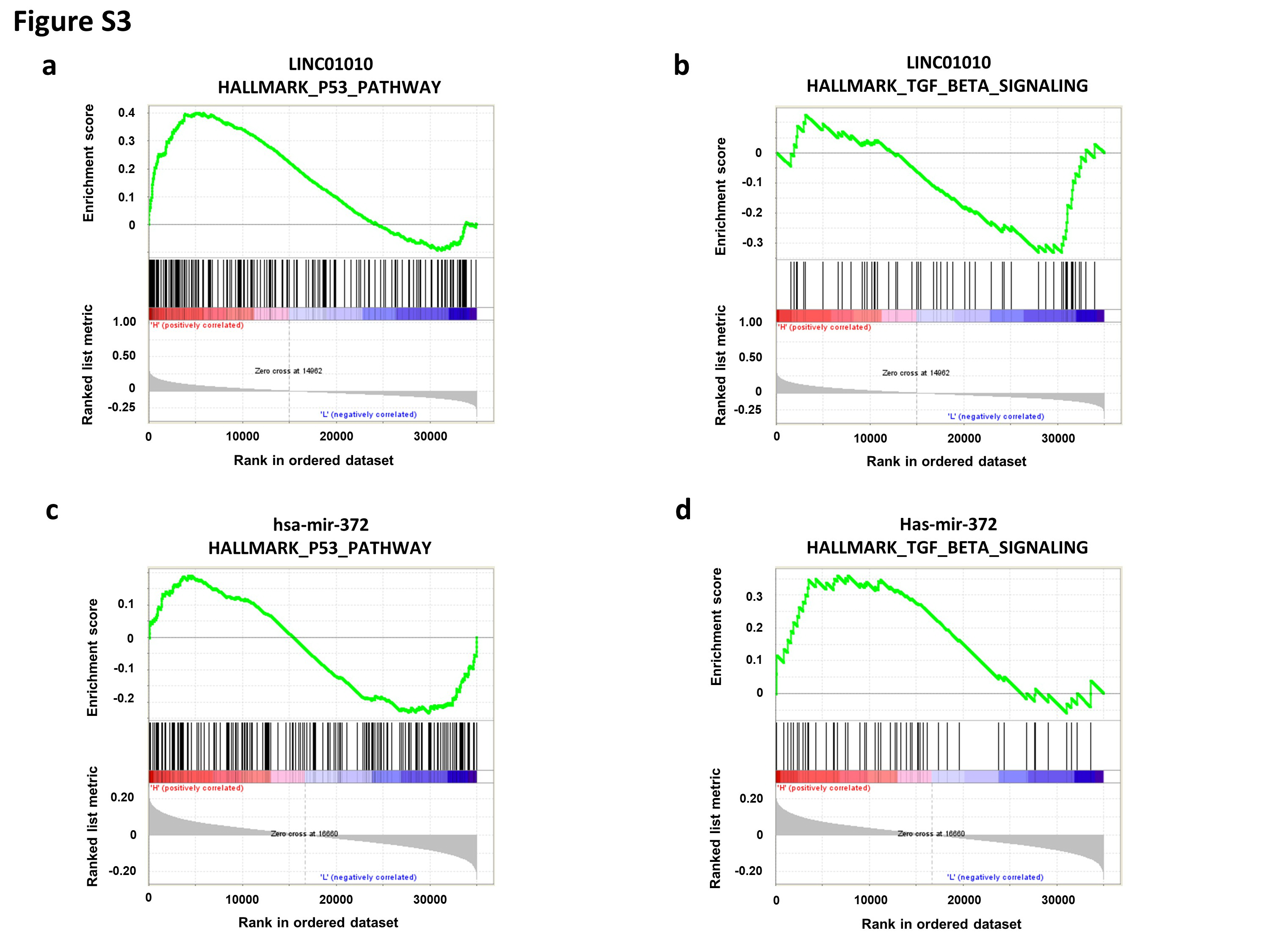

Supplement: Supplementary file 4 — Additional file 4: Fig. S3. LINC01010 and hsa-mir-372 affect the P53 pathway and the TGFβ signaling pathway. a The gene set “HALLMARK_P53_PATHWAY” was significantly enriched in high levels of LINC01010 (P < 0.0001). b The gene set “ HALLMARK_TGF_BETA_SIGNALING” was enriched in low levels of LINC01010 but no statistical difference (P = 0.12). c hsa-mir-372 and P53 pathway showed a negative correlation trend without statistical difference (P = 0.17). d The gene set “HALLMARK_TGF_BETA_SIGNALING” was significantly enriched in high levels of hsa-mir-372 (P = 0.037). [file 12935_2020_1295_MOESM4_ESM.tif]
